# The Effect of the Displacement Amplitude on the Fretting Wear of GCr15 Steel with a TiC Coating

**DOI:** 10.3390/ma15196628

**Published:** 2022-09-24

**Authors:** Xiaochu Liu, Sen He, Zhuan Zhao, Xincheng Xie, Jinrui Xiao, Zhongwei Liang

**Affiliations:** 1 Guangdong Engineering Research Centre for Strengthen Grinding and Micro/Nano High-Performance Machining, Guangzhou University, Guangzhou 510006, China; 2 School of Mechanical and Electrical Engineering, Guangzhou University, Guangzhou 510006, China; 3 School of Physics and Materials Science, Guangzhou University, Guangzhou 510006, China; 4 School of Electromechanical Engineering, Guangdong University of Technology, Guangzhou 510006, China

**Keywords:** GCr15 steel ball, fretting wear, displacement amplitude, TiC coating, mechanical ball milling

## Abstract

In the present paper, the effect of mechanical ball milling time on the fretting wear of GCr15 steel balls at different displacement amplitudes is investigated. TiC powder coating was fabricated on the surface of GCr15 steel balls using various process times, and the fretting wear tests were conducted on an AISI 52100 steel disk with the applied force of 80 N. Additionally, various displacement amplitudes (10 μm, 20 μm, and 60 μm) were selected. Specimen attributes and wear scars were characterized using an inverted metallographic microscope, a microhardness tester, an X-ray diffractometry analyzer, a white light interferometer, and a scanning electron microscope. The results showed that thick and continuous coatings could be obtained at the milling time of 18 h. The specimens processed for a longer milling time demonstrated better fretting wear resistance, which we attribute to higher microhardness of the surface layer. The coefficient of friction and wear volume of specimens at each different displacement amplitude significantly decreased with increasing milling time. As the displacement amplitude increased, the three fretting states were: partial slip coordinated by elastic deformation; partial slip state coordinated by plastic deformation; and gross slip condition. Our observations indicate that mechanical ball milling could be an efficient approach to improve the fretting wear resistance of GCr15 steel balls.

## 1. Introduction

GCr15 bearing steel is widely used in the automotive industry, the construction industry, and in the production of aerospace engines, high-speed machine tools, and wind turbines, owing to its low cost and excellent mechanical strength [1,2]. With the continuous development of different areas of industrial production, GCr15 bearing steel will be required in increasingly complex working conditions. Bearing steel materials play a vital role in the service life and performance of a product [3,4]. However, material wear caused by friction is closely related to bearing service life and operational accuracy. With GCr15 bearing steel, even slight surface wear caused by contact may provoke catastrophic failure. Therefore, bearing steel friction and wear is still an urgent problem to be solved.

Fretting wear is regarded as one of the most common causes of failure in bearing assemblies, resulting in a loosening of the fit of the steel ball with the inner ring and increasing noise and vibration. Grease hardly enters the contact area to act as a lubricant due to the small amplitude oscillating motion, resulting in the formation of dry friction on the metal surface and a rise in the coefficient of friction. Oxidative corrosion takes place on metal contact surfaces, and eventually abrasive chips are produced. Thus, the bearing loses the precision required by the host during the fretting process, accelerating bearing failure. Consequently, fretting wear has become an indispensable research direction in tribology [5]. The fretting wear characteristics of engineering materials are influenced by factors such as the combination of mating materials [6], displacement amplitude [7], normal load [7], frequency [8], surface treatment [9], surface conditions [10], and the environment [11]. It is undeniable that the displacement amplitude is one of the most fundamental factors in fretting. Displacement amplitude is usually employed to distinguish between fretting and sliding. As the displacement amplitude increases, the fretting regimes and wear damage are correspondingly different [12]. According to the different fretting damage forms, Vincent et al. [13,14] established a fretting map theory, which essentially revealed the fretting behavior and damage mechanism. The fretting map of running conditions can be classified into three characteristic regions of fretting, taking the normal force and displacement amplitude as the variables: the partial slip regime (PSR), the mixed regime (MR), and the gross slip regime (GSR) [15,16]. Zhou et al. [17] described the different fretting regimes of materials using the shapes of the $\;F_{t}-D$ curves. Xin et al. [18] assumed that the changes in the coefficient of friction and wear volume were positively correlated with an increase in the sliding displacement amplitude. Similar findings were reported by Budinski et al. [19], who found that wear grew when the hardness gap between two counterparts increased. Lin et al. [20] studied the fretting wear properties of high-nitrogen stainless steel. They found that the major wear mechanisms for bearing steel were abrasive wear and adhesion wear during the entire wear process. Zhang et al. [21] investigated the influence of the third body layer on fretting wear. At the same time, they found that the formation of a third body layer may prevent direct metal-to-metal contact, protecting the surface and reducing the wear volume. Iwabuchi et al. [22] concluded that the displacement amplitude, frequency, and other parameters might affect the overall fretting wear behavior and the removal of debris, resulting in the formation of an oxidation debris layer.

Numerous academics constantly struggle to improve the wear resistance of materials in order to increase the service life of these components. Since fretting wear is a kind of damage that takes place on the surface, surface modification is expected to enhance the strength and surface hardness of the workpiece, while a compressive residual stress field with a certain depth can be introduced into the surface layer of the workpiece. As a result, the fatigue resistance and wear resistance of materials can be significantly increased without a drastic loss of the structural properties of the material [23]. Surface modification techniques such as ultrasonic shot peening [24], laser surface treatment [25], surface mechanical attrition treatment (SMAT) [26], surface mechanical grinding treatment (SMGT) [27], surface mechanical rolling treatment (SMRT) [28], carburized treatment (CT) [29], and laser shock processing (LSP) [30] have been proven to be effective methods for refining the coarse grains of materials and generating residual compressive stresses. Furthermore, the severe plastic deformation technique can be used to refine the grain size and achieve the desired properties of the material, such as improvements in its physical properties, high hardness and strength, improved tribological properties, etc. [31]. However, the above processes are rarely applied in the surface strengthening treatments of bearing steel balls. In recent years, researchers have focused on developing and applying the mechanical ball milling technique, which is a combination of mechanical alloying and surface mechanical grinding treatment, as a surface modification. During the mechanical ball milling process, mechanical activation and deposition are triggered by repeated collisions and friction between grinding balls in a planetary ball mill, resulting in higher surface activity to promote the integration of the metal surface with the powder. The surface of the grinding balls and the metal substrate are gradually covered with powder, forming a metal film [32]. Mohammadnezhad et al. [33] deposited NiAl intermetallic layers with a nanocrystalline structure on the surface of the substrate after mechanical ball milling. They reported that a nanometer-size coating with a thickness of 470 μm was produced after ball milling for 8 h, leading to an increase in the microhardness of the substrate. Saba et al. [34] also produced TiC coatings on the surface of an AISI D2 tool steel substrate and confirmed a significant improvement in the mechanical characteristics of the substrates. Chen et al. [35] successfully prepared TiO_2_ coatings by applying Ni and TiO_2_ to the surface of ZrO_2_ ceramic balls to obtain a continuous composite coating. However, there is still insufficient research on surface modification of GCr15 steel balls based on mechanical ball milling. The influence of the strengthening treatment on the fretting wear of GCr15 steel balls has not been reported in detail so far.

The primary purpose of the research outlined in this paper was to prepare continuous hard metal coatings to improve the fretting wear resistance of GCr15 steel balls. To this end, a TiC powder coating was fabricated on GCr15 steel balls via mechanical ball milling. The effect of varying durations of ball milling on the microstructure and mechanical characteristics of GCr15 steel balls was explored using an inverted metallographic microscope, X-ray diffraction, and microhardness testing. The fretting wear tests of the GCr15 steel balls against an AISI 52,100 steel disk were performed with different displacement amplitudes. In addition, to study the wear behavior of the GCr15 steel balls, the wear scars were investigated using a white light interferometer and a scanning electron microscope.

## 2. Materials and Methods

### 2.1. Experimental Material

**Figure 1:**
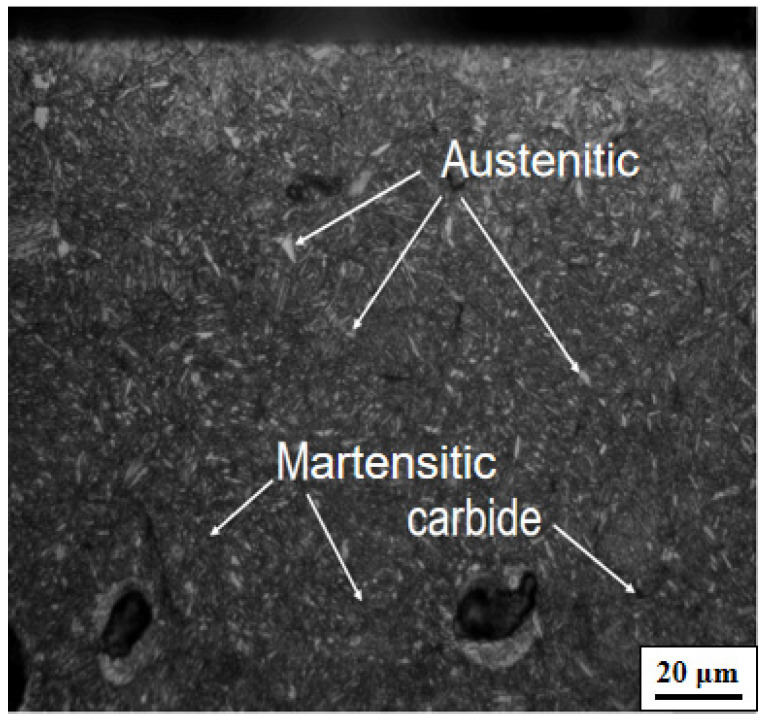
Matrix organization of GCr15 steel ball.

GCr15 bearing steel balls (Luoyang LYC bearing Co., Ltd., Luoyang, China) were prepared as the target material. The chemical composition of the GCr15 steel ball is presented in Table 1. The hardness of the material was enhanced to 700.8 HV by heat treatment. The materials were heated to 860 °C and held at that temperature for 30 min, then cooled using cold oil and tempered for 2.5 h at an ambient temperature of 160 °C, producing a tempered martensite microstructure (Figure 1).

### 2.2. Experiment

**Figure 2:**
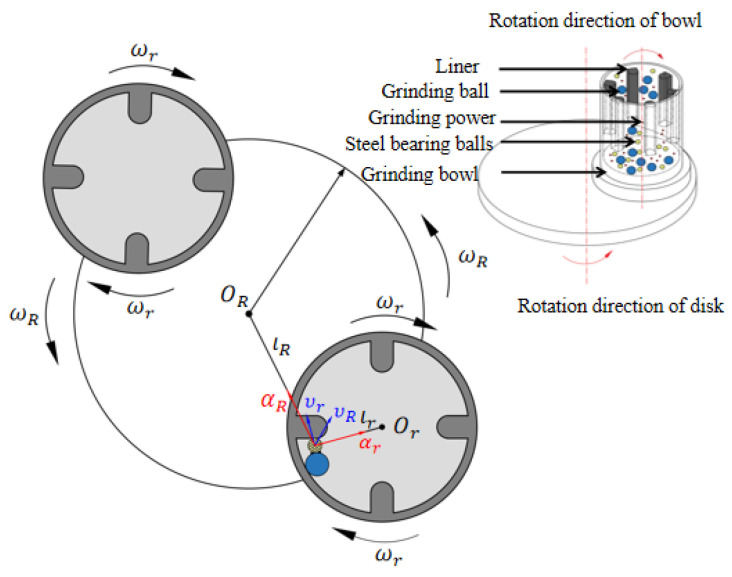
Schematic diagram of mechanical ball milling.

A powder composed of TiC with an average diameter of 48 μm and a purity of 99.5% was used in the deposition of the coatings on the surface of the steel balls and the GCr15 bearing steel balls. The average diameter of the substrate during the mechanical ball milling was 10 mm. The steel ball strengthening treatment was performed in a planetary ball mill instrument (QM-3SP2, Nanjing, China). The ball–powder weight ratio was maintained at 3:1, a total of 70 g was put into a grinding bowl with a volume of 250 mL, and the sun-disc rotation speed of the planetary ball mill was adjusted to 350 rpm for 6 h (pro. in 6 h), 12 h (pro. in 12 h), 18 h (pro. in 18 h). In order to improve the collision efficiency of the steel balls, hardened steel grinding bowls with four liners were designed and manufactured. The schematic diagram of this strengthening treatment is displayed in Figure 2. Generally, the acceleration and velocity of a ball (or particle) in the grinding bowls is influenced by its position and its interaction with another ball (or liner). In order to ensure the safety of the ball milling, the milling process was performed for 30 min with a 3-min cooling interval to avoid the overheating of the bowls. No process control agent (PCA) was applied to the bowls.

### 2.3. Characterization

The processed GCr15 steel balls were washed in an ultrasonic washer (KQ218, Suzhou, China) filled with anhydrous ethanol to remove excess powder from their surface. The surface morphology and microstructure of the cross-section of the steel balls were examined using an inverted metallographic microscope (MJ42, Guangzhou, China). A microhardness tester (HV-1000, Shanghai, China) was used to measure the hardness of the specimens under a load force of 0.49 N and a dwell time of 10 s. The phase compositions of the specimens were obtained using an X-ray diffractometry analyzer (XRD) (Rigaku SmartLab 9 Kw, Tokyo, Japan). The scanning range was set between 5°–90° with a 2°/min scan rate, and the scanning step size was 0.02° with Cu-Kα radiation at 40 kV and 30 mA.

### 2.4. Fretting Wear Tests

**Figure 3:**
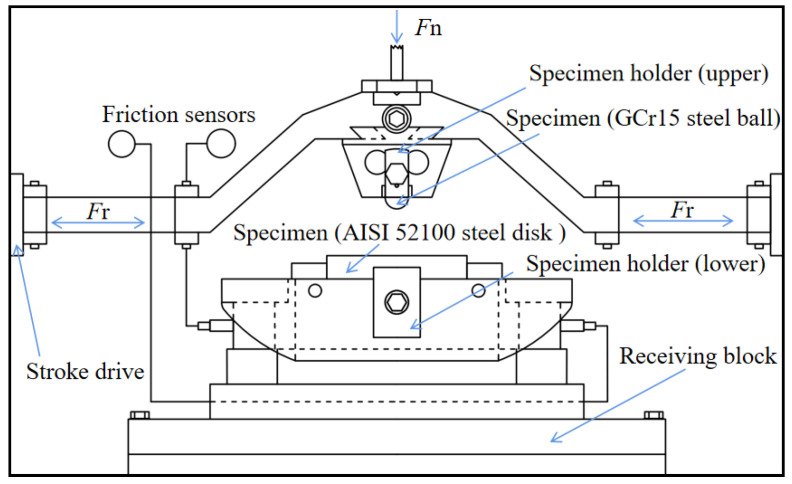
Schematic diagram of fretting wear tester.

Fretting wear tests were carried out using the commercial friction and wear tester (Optimol SRV-V, Munich, Germany). The wear tester adopted a ball-on-flat contact structure in reciprocal fretting, as shown in Figure 3. Tests were repeated three times under each condition to ensure that accurate and reliable experimental data could be obtained. The fretting tests were performed for up to 7.2 × 10^4^ cycles. The test parameters included a constant normal force of 80 N and an oscillation frequency of 20 Hz, while displacement amplitudes were set as 10 μm, 20 μm, and 60 μm, for obtaining different slip phenomena [36]. The operation was undertaken at a humidity of 35% and a temperature of 25 °C. The test specimens were GCr15 steel balls with a diameter of 10 mm, and the disk was made of AISI 52,100 steel (801.9 HV, $R_{a}<$ 0.04 μm), the dimensions of the paired part being Φ24 mm × 7.8 mm. Before and after the fretting wear tests, all of the steel balls were ultrasonically washed in anhydrous alcohol for 10 min and dried with cold air to remove unwanted matter. The wear volume and profiles of the wear scars were determined using a white light interferometer (Bruker Contour GT-1, Karlsruhe, Germany). The wear morphologies were obtained using a scanning electron microscope (SEM) (MIRA III TESCAN, Brno, Czechia).

## 3. Results and Discussion

**Figure 4:**
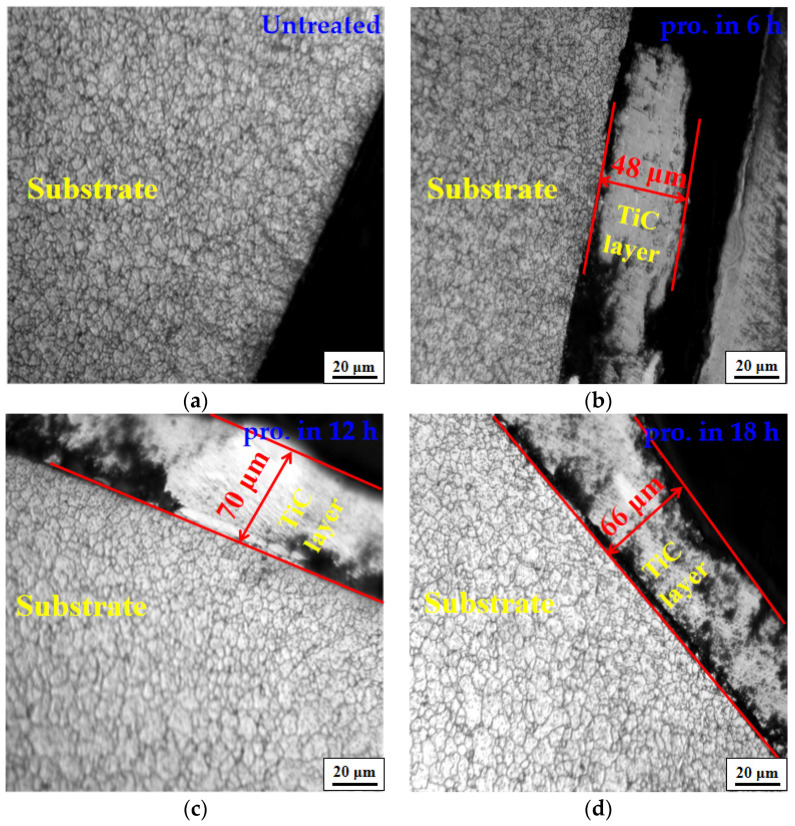
Optical microscopic photograph of cross section of GCr15 steel balls with different process times. (**a**) Untreated; (**b**) pro. in 6 h; (**c**) pro. in 12 h; (**d**) pro. in 18 h.

In order to analyze the modifications in the surface and the distribution of the coatings depending on the different ball milling times, the cross-sectional microstructures were obtained, and these are depicted in Figure 4. When the milling time came to 12 h, the average thickness of the coating reached its maximum value, after which it progressively decreased. The formation of continuous and uniform coatings was confirmed with an increased milling time. Figure 4b shows that the TiC metal powder adhered to the GCr15 steel balls with an average thickness of about 48 μm in a specimen milled for 6 h. A locally continuous but uneven coating was observed on the surface of the ball, where only a few areas were uncovered, leading to the development of the formation of dense areas of coverage on the surface of the ball. The metal particles were transferred and attached to the surface of the GCr15 steel balls by impact and friction in the beginning stages of the ball milling. Upon collision of the balls, the metal powder particles coalesced into discrete islands on the surface of the substrate after cold welding with the substrate. Extended process times increased cold welding, leading to the promotion of the thickening of the continuous metal coating. After 12 h of ball milling, a completely continuous coating with a mean thickness of approximately 70 μm could be obtained, and more metal particles were deposited, as shown in Figure 4c. As the milling time increased, the coating layer began to peel, and the thickness decreased as a result of the large amounts of lattice strain and lattice defects induced by the impacts. By increasing the processing time up to 18 h (Figure 4d), the as-milled powder coatings became more homogeneous, with a thickness size of about 66 μm. The coating and the substrate underwent plastic deformation due to ball collisions, and subsequently the powder particles and continuous metallic coatings became brittle. After milling for a certain time, the thickness of coatings reduced when the welding rate was less than the rate of fracturing. However, the coating on the substrate surface was more uniform and exhibited better extensibility compared with the former. At longer milling times, a tighter compression of the coating and firmer adherence to the surface of the substrate were achieved, due to the impact friction of the grinding balls. As a result, depositions of the surface layers occurred in a uniform manner.

**Figure 5:**
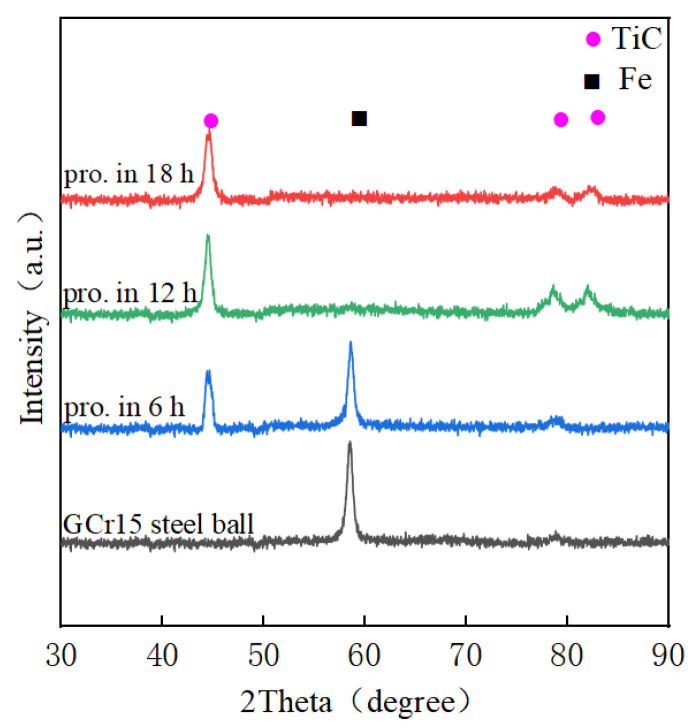
XRD patterns of TiC coatings on GCr15 steel balls after 6 h, 12 h and 18 h milling durations.

Figure 5 represents the XRD patterns of the TiC coatings on the GCr15 steel balls at various stages of milling. It is evident that the diffraction peaks of the Fe became lower, while those of the TiC became higher with an increase in processing time. This observation suggests that some TiC powder particles coalesced on the surface of the GCr15 steel balls. The diffraction peaks of the Fe could hardly be seen, and those of the TiC were still raised after 12 h ball milling, demonstrating that the almost completely continuous TiC coating adhered to the surface of the balls. During the grinding process, activation and deposition occurred simultaneously, which facilitated the bonding between the balls and the TiC metal particles. When the processing time reached 18 h, the TiC peaks gradually broadened as a result of ball milling.

**Figure 6:**
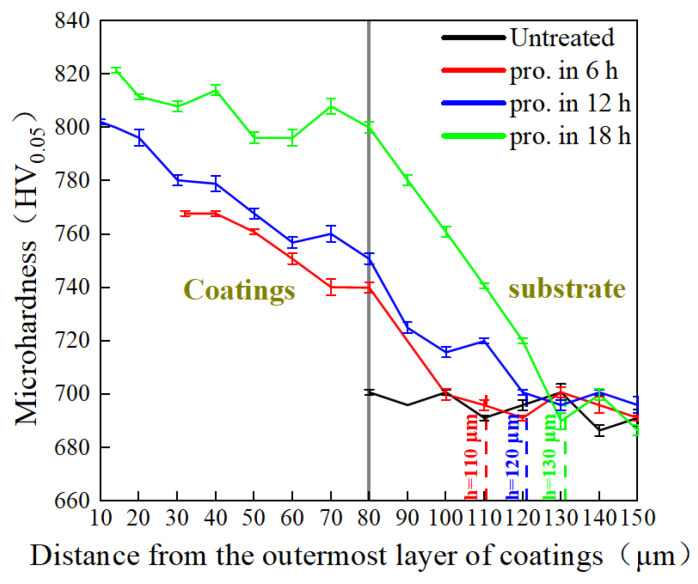
Microhardness profile of the graded coating.

The mechanical characteristics of the TiC-coated steel balls were evaluated. Figure 6 summarizes the measured results of cross-sectional hardness after different process times. The average hardness of the untreated specimens was 691.2 HV_0.05_. It can be seen from the figure that the hardness of the balls increased after the ball milling, and that the hardness increased with increased milling time. Increasing the milling duration up to 18 h led to a maximum average hardness value of 821.4 HV_0.05_. In addition, the coating showed the highest hardness in the outermost layer as a result of being subjected to most ball impacts, and it then decreased gradually towards the center of the substrate. Higher hardness of the substrate was associated with an increase in ball milling time. We attribute this enhancement to the prompt increase in strain caused by the elastic collision of the substrate with the balls as well as the strong plastic deformation layer formed by the balls grinding and colliding, and the resulting impacts on the surface of the substrate. The hardened layer of the specimens rose from 110 μm to 130 μm after ball milling, indicating that the treatment process could increase the thickness of the hardened layer of the GCr15 steel balls. The hardness of the obtained layer was higher than that produced using other metal processing techniques, such as the intermittent vacuum gas nitriding process (i.e., 120 μm) and shot peening (i.e., 85 μm) [37,38]. Crystallite size as a function of milling time is also presented in Figure 4. At longer milling times, the atomic energy gain in the surface layer increased, and the crystallite size decreased. It is worth mentioning that there was a slight gradient in the hardness curve between the coating and the substrate. This is most probably due to the harder surface resulting from grain refinement, which enhances the plastic deformation at and near the treated surface.

**Figure 7:**
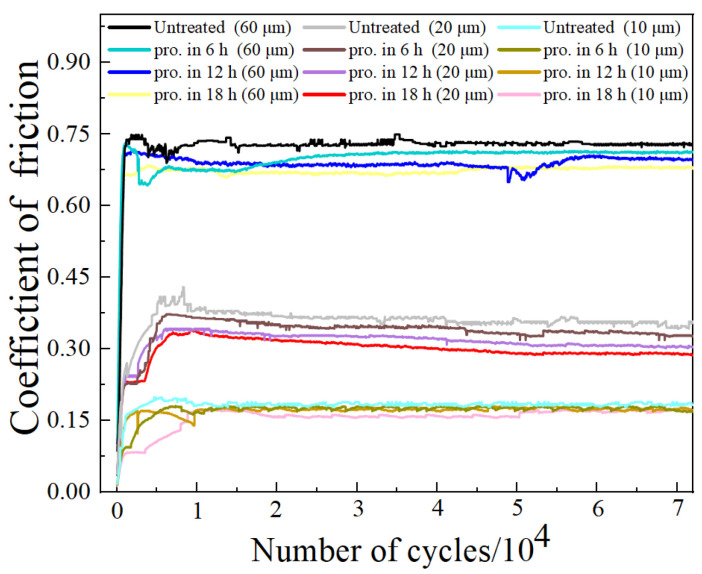
Evolution of COF for GCr15 steel balls specimens with different process times at different displacement amplitudes.

Of the data obtained from the reciprocating fretting wear process, the coefficient of friction (COF) is the most essential, as a smaller COF equate to better wear resistance. Figure 7 displays the evolution of the COF for the AISI 52,100 steel disk and the GCr15 steel balls at different displacement amplitudes and as the number of cycles increase. The COF rapidly rose to the maximum value in the initial running period (approximately 4000 wear cycles), and then maintained a relatively stable value. In the wear process, oxidation tended to occur on the surface of the substrate, generating brittle pieces of material and resulting in the formation of debris [39]. The COF decreased and oscillated significantly during the fretting wear as a consequence of the formation of third body particles [40]. A great difference was observed in the COF of the steel balls after undergoing lower milling times than the untreated balls under the same displacement amplitude. After comparing the COF data of the balls at 60 μm, it was observed that the COF decreased from 0.73 to 0.71, 0.70, and finally 0.68. This means that the COF of the balls effectively reduces with increasing process time. This was caused by the higher hardness which led to the lower COF. Moreover, the latter result may be attributed to the higher cohesion strength of the coating with an increase in milling time. As a general rule, the COF is largely influenced by the different displacement amplitudes. The COF of the balls increased when the experimental displacement amplitude rose from 10 μm to 60 μm. A minor variation took place in the COF at lower displacement amplitudes. On the other hand, the variation of the COF was much higher when the displacement amplitude reached up to 60 μm.

**Figure 8:**
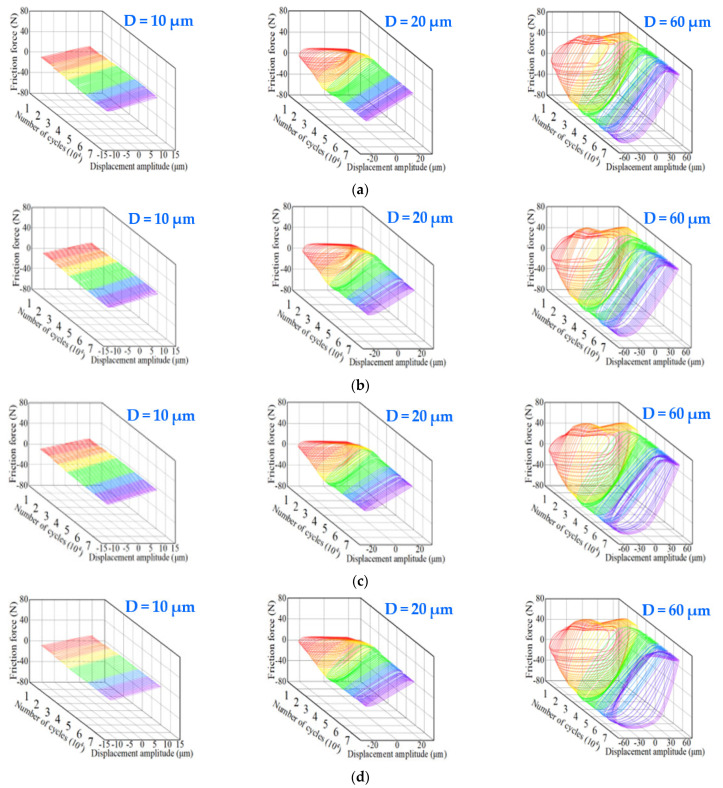
Typical $F_{t}-D-N\;$ curve maps of GCr15 steel balls at different displacement amplitudes. Different colors represent the number of cycles in different stages, with one stage for every 10,000 cycles. (**a**) Untreated; (**b**) pro. in 6 h; (**c**) pro. in 12 h; (**d**) pro. in 18 h.

**Figure 9:**
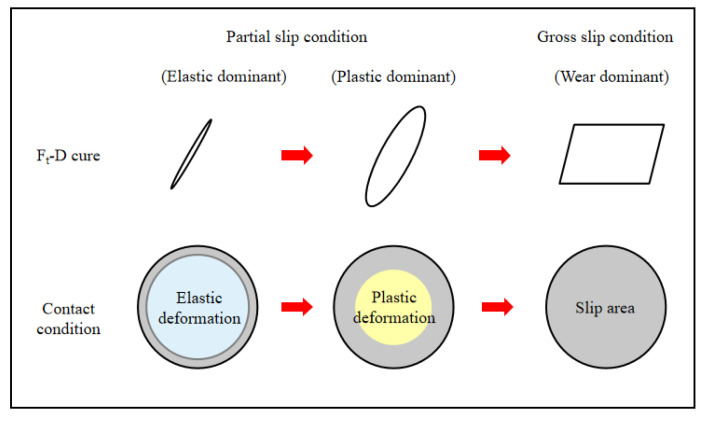
Schematic diagram of the evolution of contact states with different displacement amplitudes.

The $F_{t}-D-N$ ($F_{t}$: friction force, *D:* displacement amplitude, and *N*: number of cycles) curve is one of the most critical indicators obtained from the tribological tests, and it describes the damage mechanisms and fretting regimes of the wear surfaces in contact with each other during the fretting process. Figure 8 shows the $F_{t}-D-N$ curve maps of the GCr15 steel balls before and after treatment at different displacement amplitudes. The $\;F_{t}-D-N$ curve appeared approximately linear during the entire test at the displacement amplitude of 10 μm, indicating that the fretting contact region of the specimen was in a state of partial slip coordinated by elastic deformation. When the displacement amplitude was 20 μm, the $F_{t}-D-N$ curve initially had an elliptical shape, but then transformed and became linear. This indicates that the fretting contact region was in a partial slip state coordinated by plastic deformation. The $\;F_{t}-D-N$ curve was basically a quasi-parallelogram at 60 μm, consistent with the gross slip condition of the fretting contact region. The representative $F_{t}-D-N$ curve of the balls after 18 h of treatment formed a parallelogram (Figure 8b) at 60 μm due to the low coefficient of friction of the balls after milling. A schematic diagram of the evolution of the contact states with different displacement amplitudes, according to the results of the $\;F_{t}-D-N$ curve maps, is presented in Figure 9 [36]. The figure indicates that the partial slip condition transformed to the gross slip condition when the displacement amplitude increased from 10 μm to 60 μm.

**Figure 10:**
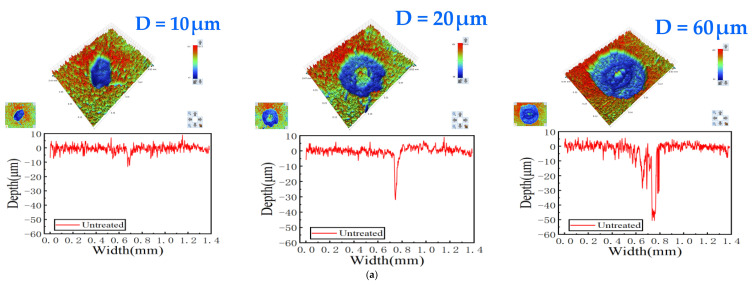

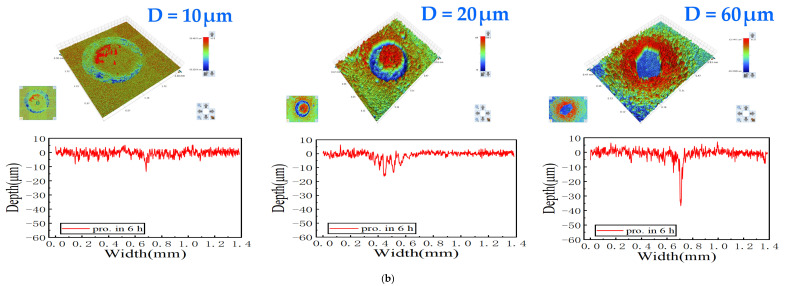

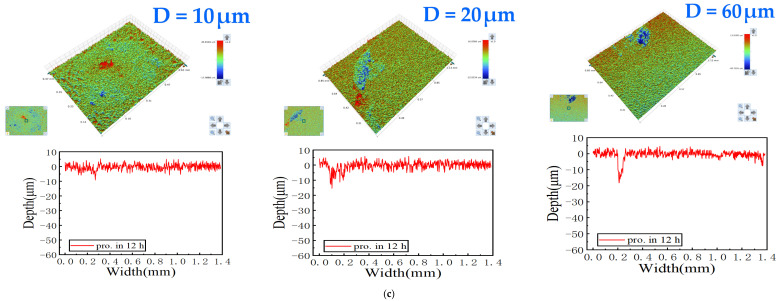

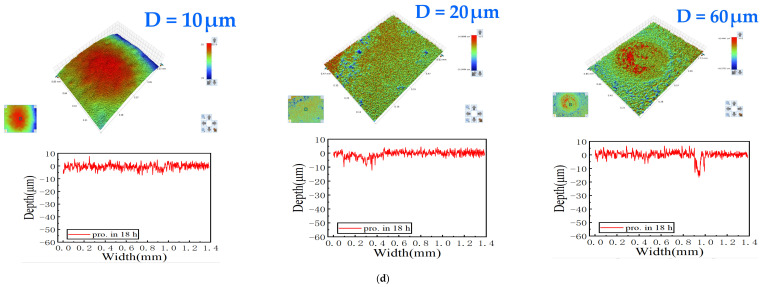
Two-dimensional/three-dimensional morphology of wear scar morphologies on balls after various process times and at different displacement amplitudes. (**a**) Untreated; (**b**) pro. in 6 h; (**c**) pro. in 12 h; (**d**) pro. in 18 h.

The three-dimensional optical profilometry images of all the wear scars on the balls before and after treatment at different displacement amplitudes are displayed in Figure 10. The image displays the number of grooves on the ball surface after the wear tests, and the colors indicate the depth of the wear scar in each area, according to the scale. The greatest degree of wear generally existed in the central part of the wear scar. During the wear process, wear debris was squeezed out of the wear pits and accumulated on the contact edges of the wear region [41]. We observed that the maximum wear depth increased from 15 μm to 50 μm with the rise of the displacement amplitude from 10 μm to 60 μm (Figure 10a). When the displacement amplitude was less than 20 μm, the wear scar was partially raised, with minor wear damage and residual wear debris in the crater. The wear profile was a regular elliptical shape at a displacement amplitude of 60 μm, which was due to the fact that wear debris was discharged more easily from the wear scale.

**Figure 11:**
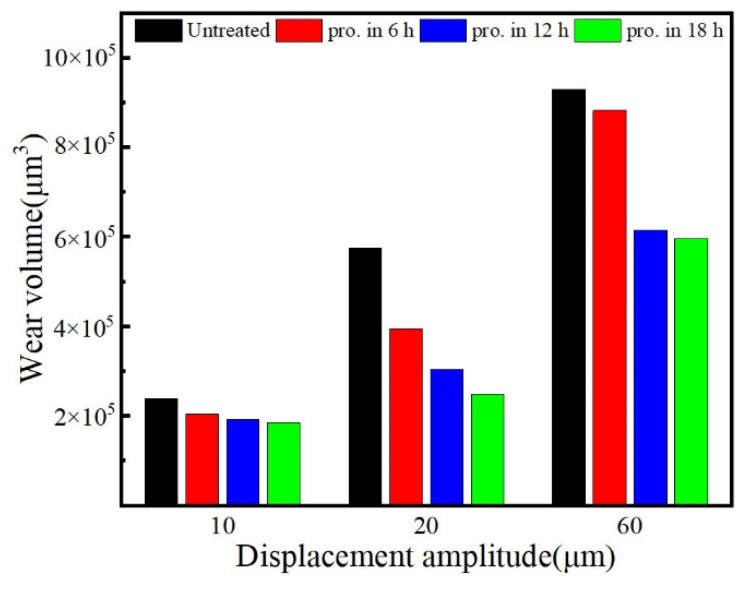
Wear volume of wear scars on balls after various process times and at different displacement amplitudes.

Wear volume is a critical indicator for assessing the fretting wear resistance of metals. A statistics chart for the wear volumes at different displacement amplitudes is presented in Figure 11. At a displacement amplitude of 60 μm, the wear volumes of the untreated balls and the balls processed for 6 h, 12 h, and 18 h, were approximately 9.2 × 10^5^, 8.8 × 10^5^, 6.1 × 10^5^, and 5.9 × 10^5^ μm^3^, respectively. It can be seen that the balls processed by mechanical ball milling may exhibit better tribological performance. In general, greater hardness is accompanied by better tribological behaviors by the balls. Therefore, the wear volume can be assumed to decrease with increasing steel ball process time.

**Figure 12:**
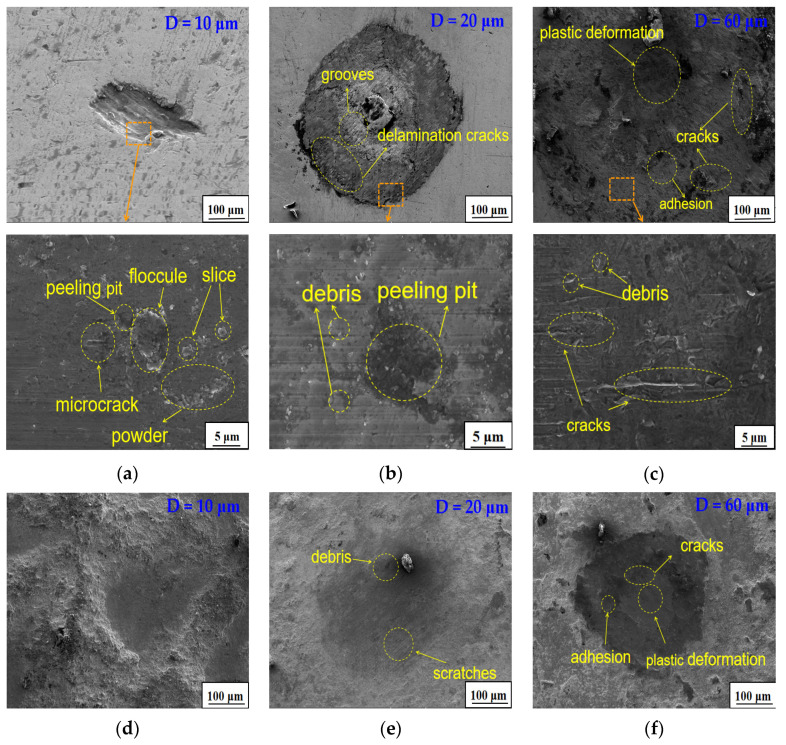
Representative SEM results of wear surfaces on GCr15 steel balls at different displacement amplitudes. (**a**) Untreated, D = 10 μm; (**b**) untreated, D = 20 μm; (**c**) untreated, D = 60 μm; (**d**) pro. in 18 h, D = 10 μm; (**e**) pro. in 18 h, D = 20 μm; (**f**) pro. in 18 h, D = 60 μm.

For a better analysis of the wear mechanism, representative SEM graphs of wear surfaces on the GCr15 steel balls at different displacement amplitudes in the fretting wear process are given in Figure 12. We noted that only slight scratches appeared on the surface of the ball at a displacement amplitude of 10 μm (Figure 12a). There are three types of wear debris in a fretting wear scar: slice, powder, and floccule. In the contact process, hard slice debris was created as a result of the breakage of both the wear material and the oxidation layers [42]. The slice debris was gathered into floccule debris and transformed into powder debris under repeated tumbling and vibration wear. Additionally, a few fine microcracks and peeling pits appeared on the wear scars. At a displacement amplitude of 20 μm, the fretting wear operated in a partial slip regime, which was indicated by a few dislodged particles, i.e., third body material, covering the contact area. Additionally, the joined asperities in the center of the adhesion region were plastically sheared along the fretting direction. The loose third body material was transported away from the contact area and accumulated at the edge of the wear scar. The delamination cracks and plow grooves at the edge of the wear scar can be seen in Figure 12b. When the displacement amplitude was increased up to 60 μm, adhesion and plastic deformations were found on the surface due to the steel ball being submitted to shear and normal stress (Figure 12c). The material was usually plastically deformed by stacking, owing to the sliding contact [43]. The craters generated by peeling expanded the accumulation space for loose wear particles, with the result that most of the wear scars on the surface were covered by a thick wear debris layer. This may be because the width of the wear scar and the amount of wear debris generally increased with an increase in the displacement amplitude. The wear profile of the ball was processed in 18 h at different displacement amplitudes, as observed in Figure 12d–f. A comparison of the GCr15 steel balls before and after treatment showed that the wear surface of the processed ball was relatively rough and did not form a complete wear track at 10 μm (Figure 12d). Minor damage was observed in the central area of the friction. Some relatively slight scratches and few pieces of wear debris in the process of wear and tear were observed at a displacement amplitude of 20 μm (Figure 12e). The number of cracks and the amount of stacking materials on the surface of the processed ball were less than those observed in the untreated ball at the same displacement amplitude. This could be explained by the higher surface hardness of the ball after milling, and the fact that the hardness gap between the ball and the steel disk was reduced. Even at higher displacement amplitudes, relatively minor adhesion and plastic deformation was observed on the processed ball surface. The particles flaked very little and accumulated in the pit on the wear scar due to the high surface strength, which reduced the degree of material wear. We are certain that increased ball milling time leads to reduced fretting wear damage and an increase in the hardness of the substrate.

## 4. Conclusions 

In this w ork, the effect of mechanical ball milling time on the fretting wear of GCr15 steel balls at different displacement amplitudes was investigated. Additionally, the effect of ball milling time on the coating thickness, surface hardness, and fretting wear resistance of the GCr15 steel balls was investigated.

GCr15 steel balls can obtain a coating with a thickness of 70 μm and a hardness of 821.4 HV_0.05_ in the grinding bowl with four liners. The thickness of the hardened layer can be over 130 μm. At a displacement amplitude of 60 μm, the COF decreases from its original 0.73 to 0.71, 0.70, and 0.68 following processing times of 6 h, 12 h, and 18 h, respectively.

The fretting wear mechanisms of steel balls are mainly adhesive wear and abrasive wear. With a processing time of 18 h, the wear loss was effectively reduced by 22%, 56%, and 35% at displacement amplitudes of 10 μm, 20 μm, and 60 μm, respectively. After mechanical ball milling, the improved fretting wear resistance of the GCr15 steel balls is obvious, and we attribute this to a reduction in the number of chips, cracks, and peeling pits. This investigation offers an alternative method to improve the service life of bearing balls.

## Figures and Tables

**Table 1:** Actual chemical composition (wt%) of GCr15 steel balls used during experiment.

C	Cr	Si	Mn	Mo	Cu	Ni	P	S	Fe
1.00	1.51	0.22	0.30	0.05	0.08	0.18	0.003	0.002	Bal.

## Data Availability

The data presented in this study are available in the article.

## References

[1] Xue Y., Shi X., Zhou H., Lu G., Zhang J. (2020). Effects of groove-textured surface combined with Sn–Ag–Cu lubricant on friction-induced vibration and noise of GCr15 bearing steel. Tribol. Int..

[2] Cao Y.J., Sun J.Q., Ma F., Chen Y.Y., Cheng X.Z., Gao X., Xie K. (2017). Effect of the microstructure and residual stress on tribological behavior of induction hardened GCr15 steel. Tribol. Int..

[3] Buling A., Sändker H., Stollenwerk J., Krupp U., Hamann-Steinmeier A. (2016). Laser surface pretreatment of 100Cr6 bearing steel—Hardening effects and white etching zones. Appl. Surf. Sci..

[4] Roy S., Sundararajan S. (2016). The effect of heat treatment routes on the retained austenite and Tribomechanical properties of carburized AISI 8620 steel. Surf. Coat. Technol..

[5] Waterhouse R.B. (1984). Fretting wear. Wear.

[6] Blanchard P., Colombie C., Pellerin V., Fayeulle S., Vincent L. (1991). Material effects in fretting wear: Application to iron, titanium, and aluminum alloys. Metall. Trans. A.

[7] Gaspar M.C., Ramalho A. (2002). Fretting behaviour of galvanised steel. Wear.

[8] Söderberg S., Bryggman U., Mccullough T. (1986). Frequency effects in fretting wear. Wear.

[9] Prakash B., Ftikos C., Celis J.P. (2002). Fretting wear behavior of PVD TiB2 coatings. Surf. Coat. Technol..

[10] Sato J., Shima M., Sugawara T., Tahara A. (1988). Effect of lubricants on fretting wear of steel. Wear.

[11] Mohrbacher H., Blanpain B., Celis J.P., Roos J.R. (1995). The influence of humidity on the fretting behaviour of PVD TiN coatings. Wear.

[12] Zhu M.H., Zhou Z.R. (2011). On the mechanisms of various fretting wear modes. Tribol. Int..

[13] Vincent Z. (1993). Effect of external loading on wear maps of aluminium alloys. Wear.

[14] Fouvry S., Kapsa P., Vincent L. (1995). Analysis of sliding behaviour for fretting loadings: Determination of transition criteria. Wear.

[15] Vingsbo O., Söderberg S. (1988). On fretting maps. Wear.

[16] Leonard B.D., Sadeghi F., Shinde S., Mittelbach M. (2012). A Numerical and Experimental Investigation of Fretting Wear and a New Procedure for Fretting Wear Maps. Tribol. Trans..

[17] Zhou Z.R., Vincent L. (1995). Mixed fretting regime. Wear.

[18] Xin L., Wang Z.H., Li J., Lu Y.H., Shoji T. (2016). Fretting Wear Behavior and Mechanism of Inconel 690 Alloy Related to the Displacement Amplitude. Tribol. Trans..

[19] Budinski, Kenneth G. (2013). Effect of hardness differential on metal-to-metal fretting damage. Wear.

[20] Lin H., Yang M.-s., Shu B.-p. (2020). Fretting wear behaviour of high-nitrogen stainless bearing steel under lubrication condition. J. Iron Steel Res. Engl. Ed..

[21] Zhang S., Liu L., Ma X., Zhu G., Tan W. (2022). Effect of the third body layer formed at different temperature on fretting wear behavior of 316 stainless steel in the composite fretting motion of slip and impact. Wear.

[22] Iwabuchi A. (1991). The role of oxide particles in the fretting wear of mild steel. Wear.

[23] Wang X.Y., Li D.Y. (2003). Mechanical, electrochemical and tribological properties of nano-crystalline surface of 304 stainless steel. Wear.

[24] Liu G., Lu J., Lu K. (2000). Surface nanocrystallization of 316L stainless steel induced by ultrasonic shot peening—ScienceDirect. Mater. Sci. Eng. A.

[25] Mondal A.K., Kumar S., Blawert C., Dahotre N.B. (2008). Effect of laser surface treatment on corrosion and wear resistance of ACM720 Mg alloy. Surf. Coat. Technol..

[26] Bahl S., Suwas S., Ungàr T., Chatterjee K. (2017). Elucidating microstructural evolution and strengthening mechanisms in nanocrystalline surface induced by surface mechanical attrition treatment of stainless steel. Acta Mater..

[27] Li W.L., Tao N.R., Lu K. (2008). Fabrication of a gradient nano-micro-structured surface layer on bulk copper by means of a surface mechanical grinding treatment. Scr. Mater..

[28] Carneiro L., Wang X., Jiang Y. (2020). Cyclic deformation and fatigue behavior of 316L stainless steel processed by surface mechanical rolling treatment. Int. J. Fatigue.

[29] Izciler M., Tabur M. (2006). Abrasive wear behavior of different case depth gas carburized AISI 8620 gear steel. Wear.

[30] Lu J.Z., Luo K.Y., Zhang Y.K., Cui C.Y., Sun G.F., Zhou J.Z., Zhang L., You J., Chen K.M., Zhong J.W. (2010). Grain refinement of LY2 aluminum alloy induced by ultra-high plastic strain during multiple laser shock processing impacts—ScienceDirect. Acta Mater..

[31] Estrin Y., Vinogradov A. (2013). Extreme grain refinement by severe plastic deformation: A wealth of challenging science. Acta Mater..

[32] Takacs L. (2007). Preparation of Coatings by Mechanical Alloying. Chem. Sustain. Dev..

[33] Mohammadnezhad M., Shamanian M., Enayati M.H., Salehi M. (2013). Influence of annealing temperature on the structure and properties of the nanograined NiAl intermetallic coatings produced by using mechanical alloying. Surf. Coat. Technol..

[34] Saba F., Raygan S., Abdizadeh H., Dolatmoradi A. (2013). Preparing TiC coating on AISI D2 steel using mechanical milling technique. Powder Technol..

[35] Chen L., Chen H., Li R., He W., Yang Y. (2021). Fabrication of Nickel Coatings on Zirconia Balls by Mechanical Ball Milling and the Process Analysis. Mater. Sci..

[36] Xinlu Y., Gen L., Xiaoyu Z. (2021). Effect of Displacement Amplitude on Fretting Wear Behavior of Copper Magnesium Alloy. Tribology.

[37] Zhang C., Fu T., Chen H., Gao Y. (2021). Microstructure Evolution of Surface Gradient Nanocrystalline by Shot Peening of TA17 Titanium Alloy. Metall. Mater. Trans. A.

[38] Yang C., Ma Y., Liu J., University G.N. (2019). Microstructure and Wear Resistance of Intermittent Vacuum Gas Nitriding Layer on TB8 Titanium Alloy. Rare Met. Mater. Eng..

[39] Rai P.K., Shekhar S., Nakatani M., Vajpai S.K., Ota M., Ameyama K., Mondal K. (2018). Effects of grain size gradients on the fretting wear of a specially-processed low carbon steel against AISI E52100 bearing steel. Wears.

[40] Baydoun S., Fouvry S., Descartes S., Arnaud P. (2019). Fretting wear rate evolution of a flat-on-flat low alloyed steel contact: A weighted friction energy formulation. Wear.

[41] Xin L., Lu Y., Shoji T. (2017). The role of material transfer in fretting wear behavior and mechanism of Alloy 690TT mated with Type 304 stainless steel. Mater. Charact..

[42] La P.Q., Ma J.Q., Zhu Y.T., Yang J., Liu W.M., Xue Q.J., Valiev R.Z. (2005). Dry-sliding tribological properties of ultrafine-grained Ti prepared by severe plastic deformation. Acta Mater..

[43] Tong Z.P., Ren X.D., Zhou W.F., Adu-Gyamfi S., Chen L., Ye Y.X., Ren Y.P., Dai F.Z., Yang J.D., Li L. (2018). Effect of laser shock peening on wear behaviors of TC11 alloy at elevated temperature. Opt. Laser Technol..

